# Climate Change Impacts on Plant-Parasitic Nematodes in Agroecosystems

**DOI:** 10.3390/pathogens15040425

**Published:** 2026-04-14

**Authors:** Refik Bozbuğa, Furkan Ulaş, Özlem Urtekin, Muhammad Aasim, Mustafa İmren, Rachid Lahlali, Muhammad Amjad Ali, Fouad Mokrini, Abdelfattah Dababat

**Affiliations:** 1 Department of Plant Protection, Faculty of Agriculture, Eskisehir Osmangazi University, Eskisehir 26160, Türkiye; refik.bozbuga@ogu.edu.tr (R.B.); ozlemurtekin@gmail.com (Ö.U.); 2 Department of Plant Protection, Faculty of Agricultural Sciences and Technologies, Sivas University of Science and Technology, Sivas 58010, Türkiye; furkanulas@sivas.edu.tr; 3 Department of Precision Agriculture and Agricultural Robotics, Faculty of Agricultural Sciences and Technologies, Sivas University of Science and Technology, Sivas 58010, Türkiye; maasim@sivas.edu.tr; 4 Department of Plant Protection, Faculty of Agriculture, Bolu Abant Izzet Baysal University, Bolu 14030, Türkiye; mustafaimren@ibu.edu.tr; 5 Phytopathology Unit, Department of Plant Protection, Ecole Nationale d’Agriculture de Meknès, Km10, Rte Haj Kaddour, BP S/40, Menkes 50001, Morocco; rlahlali@enameknes.ac.ma; 6 Department of Plant Pathology, University of Agriculture, Faisalabad 38040, Pakistan; amjad.ali@uaf.edu.pk; 7 Nematology Laboratory, Biotechnology Research Unit, National Institute of Agricultural Research, INRA-Rabat, Avenue Ennasr, BP 415 Rabat Principale, Rabat 10090, Morocco; fmokrini.inra@gmail.com; 8 International Maize and Wheat Improvement Centre (CIMMYT), Ankara 06170, Türkiye; 9 School of Agriculture, The University of Jordan, Amman 11942, Jordan

**Keywords:** climate change, plant-parasitic nematodes, temperature increase, atmospheric CO_2_, nematode-host interactions

## Abstract

Climate change significantly impacts agricultural ecosystems through rising temperatures, changing precipitation patterns, increasing atmospheric CO_2_ levels, and more frequent extreme weather events. These environmental changes have a pronounced effect on plant-parasitic nematodes (PPNs; phylum Nematoda), which cause serious crop losses on a global scale. This review aims to provide a comprehensive evaluation of current knowledge on how major climate change drivers influence the biology, population dynamics, host–plant interactions, and geographic distribution of PPNs in agricultural systems. Recent studies show that rising temperatures accelerate nematode development, increasing the number of generations within a production season and facilitating the spread of many economically important species toward higher latitudes and elevations. Changes in precipitation patterns and soil moisture directly affect nematode survival, mobility, and infection success, and these effects often vary depending on regional conditions and nematode species. Elevated atmospheric CO_2_ levels modify plant–nematode interactions by increasing root biomass, altering rhizosphere processes, and regulating plant defense pathways (e.g., jasmonic acid and salicylic acid signaling), which may enhance host susceptibility and infection intensity. Furthermore, extreme climate events can disrupt the natural balance in soil ecosystems, weakening natural antagonist–nematode relationships. However, responses of PPNs to climate change are not uniform, and contrasting findings across studies indicate that these responses are strongly shaped by species-specific traits and environmental variability. In addition, future research should focus on long-term and multi-factorial field studies to better capture the combined effects of climate drivers. Overall, climate change is expected to increase PPN prevalence and drive shifts in their geographic distribution, highlighting the need for climate-sensitive and regionally adapted nematode management strategies.

## 1. Introduction

Global warming, driven by increasing greenhouse gas emissions, industrial activities, and changes in atmospheric composition has emerged as a major environmental challenge, resulting in rising global temperatures and more frequent extreme climate events such as droughts, floods, and heatwaves [1,2,3]. These changes generally affect the overall agro-ecosystem, but specifically pose strong effects on soil ecosystems, where alterations in temperature and moisture regimes directly influence belowground biological processes [4].

Nematodes are microscopic, unsegmented roundworms that are widely distributed in soil environments. They are a crucial part of soil ecosystems, playing key roles in the decomposition of organic matter, regulation of the nutrient cycle, and balance of microbial communities [5]. However, plant-parasitic nematodes (PPNs) disrupt root morphology by creating feeding sites in plant roots, limiting water and nutrient uptake, and negatively affecting plant development [6,7].

Given these characteristics, PPNs represent a key model group for assessing the effects of climate change in agricultural ecosystems. Globally, numerous economically important plant species, ranging from cereals to vegetables, fruit and ornamental plants to forest ecosystems, are exposed to PPN attacks, leading to significant losses in agricultural production [8]. Today, approximately 4300 identified PPN species are commonly found in open fields and protected production systems, and the annual global crop loss caused by these species is estimated to be over US$150 billion [7,9].

The most economically important PPN genera include *Meloidogyne*, *Heterodera*, *Globodera*, and *Pratylenchus*, which impose significant physiological stress in plants by forming special structures such as galls in the host plant or by directly damaging root tissues [7]. These galls have several giant cells and syncytia which act as feeding sites for the sedentary nematodes. The distribution, density, and extent of crop losses caused by the PPNs are determined by the interaction of PPNs with host plant species, agricultural practices, soil properties, and environmental conditions [10]. Abiotic factors, particularly temperature, soil moisture, and rainfall patterns, directly affect the survival, reproduction, and host-finding abilities of nematodes [11,12].

Therefore, environmental factors associated with climate change can significantly alter the species composition, host interactions, and geographic distribution of PPN communities [13]. PPNs have high plasticity towards the changing environmental conditions. This plasticity is the function of several survival mechanisms such as anhydrobiosis, cryptobiosis, cyst formation, and dormancy, which enable the PPNs to persist under adverse conditions such as drought, extreme heat, and flooding [14]. These adaptive traits allow PPNs to pose a persistent threat to agricultural systems even under climate change scenarios [15].

Recent studies have shown that the effects of climate change on PPN populations vary depending on regional and ecological conditions [16,17]. While the abundance of some species increases, the distribution ranges of others are shrinking or shifting towards higher latitudes and altitudes [18]. Furthermore, rising temperatures accelerate the life cycle of nematodes, allowing them to produce more generations within a single growing season; this increases the intensity of plant–nematode interactions, leading to greater agricultural losses [19].

This review provides a comprehensive and critical evaluation of how major climate change drivers, including temperature, precipitation, atmospheric CO_2_, and extreme events, influence plant–nematode interactions in agricultural systems. The existing literature indicates limitations in addressing these effects in an integrated manner. Accordingly, the objectives of this review are to: (i) evaluate the effects of climate change drivers on the biology and population dynamics of PPNs, (ii) analyse plant–nematode interactions under changing environmental conditions, and (iii) provide recommendations for the sustainable management of PPNs under climate change.

## 2. Global Warming and Plant-Parasitic Nematodes in Agroecosystems

One of the most significant effects of global warming on agricultural ecosystems is the increase in temperature, which directly affects the biological processes of PPNs [20]. As ectothermic organisms, nematodes’ metabolic rates, growth rates, and life cycle durations are tightly linked to temperature in the surroundings [21]. Temperatures exceeding the optimal range cause an increase in egg hatching rates, accelerated larval development, and the formation of more generations of nematodes within the same production season [17]. This situation significantly increases the damage potential of economically important PPN species, particularly those belonging to the genera, *Meloidogyne*, *Heterodera*, *Globodera*, and *Pratylenchus*. However, the effects of global warming are not limited to temperature increases; they are better understood holistically when considered alongside other climatic components.

One important consequence of elevated temperatures is the observed expansion in the geographic distribution ranges of PPNs. Many species previously found only in tropical and subtropical regions are now reported to be detected in temperate climate zones and higher altitude areas [22]. Ecological observations in recent years have revealed that species such as *Meloidogyne incognita* and *M. javanica* are shifting northward and establishing permanent populations in regions where they were not previously reported [23]. This geographical expansion, accompanied by adaptation to new host plants, increases the risk to agricultural production on a broader scale [19].

**Figure 1:**
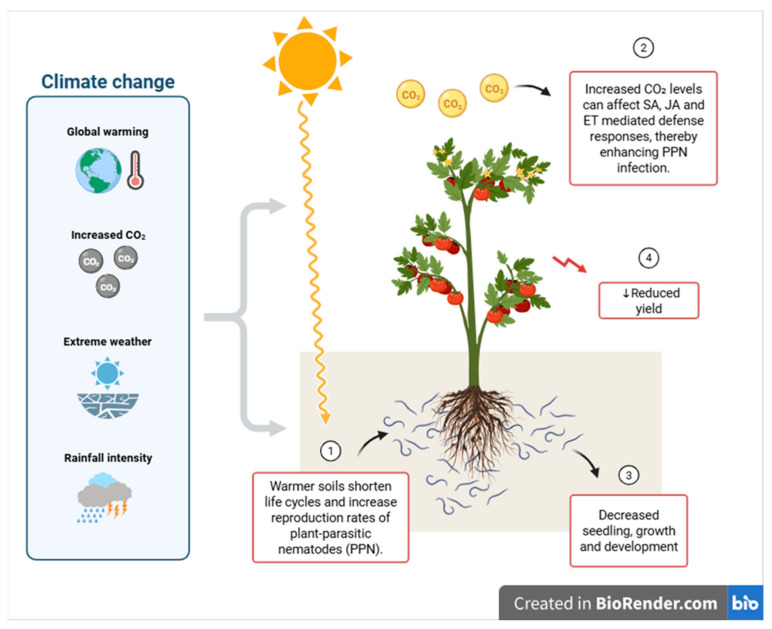
Conceptual overview of climate change impacts on plant-parasitic nematodes in agricultural systems.

Global warming is not limited to temperature increases, but is also attributed to significant changes in precipitation patterns and soil moisture dynamics [24]. Disruptions in rainfall patterns and increased periods of drought can limit the mobility and infection success of nematodes in the soil due to reduced soil moisture [13]. However, some PPN species can survive for long periods under unfavourable conditions because of physiological adaptation mechanisms such as anhydrobiosis and can quickly become active when conducive environmental conditions are restored [25,26]. Under excessive rainfall conditions, soil water saturation and oxygen depletion occur; this situation suppresses the development of some species while creating advantageous ecological niches for others. In this context, the effects of rainfall changes on PPN populations vary greatly depending on the species, soil structure, and regional climate characteristics [6]. These multi-layered interactions are evaluated within the framework of the interrelationships between plants, soil properties, microbial communities, and soil fauna (Figure 1).

One of the indirect effects of global warming on agricultural systems is the increase in atmospheric CO_2_ concentration. Rising CO_2_ levels alter plant physiological processes, increasing photosynthesis rates, leading to root biomass expansion and increased carbon flux in the rhizosphere [27]. Root system development can increase infection severity by creating larger feeding areas for some PPN species. Additionally, it has been reported that CO_2_ increase affects plant defense mechanisms, particularly by causing changes in defense pathways such as jasmonic acid and salicylic acid, which may increase plant susceptibility to nematode infections [28]. These physiological changes are supported by experimental studies showing that *Meloidogyne* species exhibit more aggressive infection profiles under high CO_2_ conditions [29].

Increases in the frequency and intensity of extreme weather events also significantly affect PPN ecology. Sudden heat waves, fires, floods, and severe droughts can disrupt soil structure, leading to sharp fluctuations in the populations of both nematodes and their natural enemies [22]. In particular, fires can drastically alter the composition of the nematode fauna by reducing organic matter in the topsoil and suppressing biological activity [15]. Similarly, floods can cause high mortality in aerobic nematode species by creating hypoxic conditions in the soil [30]. However, it is also known that some PPN species can recover quickly after such stresses and become dominant again in a short period of time [30].

Overall, the effects of global warming on PPN ecology are noticeable as nonlinear, species-specific, and context-sensitive complex responses [22]. While some species benefit from changing climate conditions by expanding their distribution ranges and increasing their pest potential, others are negatively affected and show declines in their populations [19]. However, many PPN species of critical importance to agricultural production have expanded their ecological suitability ranges globally, increased their virulence, and made nematode management increasingly complex [17].

## 3. Key Climate Change Drivers Affecting Plant-Parasitic Nematodes

### 3.1. Rising Temperature and Thermal Responses of Plant-Parasitic Nematodes

Rising global temperatures are emerging as one of the most decisive climatic drivers, shaping the biology, population dynamics, and ecological suitability ranges of PPNs [20]. In nematodes, which are ectothermic organisms, metabolic rate, development time, and reproductive capacity are directly dependent on environmental temperature [31]. Therefore, increases in temperature significantly affect PPN egg hatching rates, larval mobility, host plant entry rates, and female nematode egg production capacity [32]. When the optimal temperature range is exceeded, the acceleration of metabolic processes shortens the life cycle of nematodes and allows them to produce more generations within a production season [17]. For example, the development time of *Meloidogyne incognita* is approximately 28 days at 20 °C, but drops to 14 days at 30 °C, clearly demonstrating the potential of this species to multiply rapidly in agricultural areas as temperatures rise [33].

Temperature changes not only affect the development speed but also the behavioural and infection-related characteristics of nematodes. Since the amount and composition of volatile organic compounds released from root tips change depending on temperature, the success of PPNs in detecting host roots and orienting towards them is also affected by this process [16]. While high temperatures increase root penetration and infection success in some species, exceeding certain thresholds can cause protein denaturation and enzymatic degradation, which can increase mortality in nematodes [17]. These physiological and behavioural responses play a decisive role in the long-term ecological distribution and pest profiles of nematodes.

Temperature increases associated with climate change also cause significant shifts in the geographical distribution and ecological behaviour of PPNs. Studies indicate that rising temperatures accelerate the spread of wood-damaging nematode species such as *Bursaphelenchus xylophilus* and their vector insects (e.g., *Monochamus alternatus*) toward northern latitudes [34]. This process is causing an increase in the spread and severity of pine wilt disease in some regions of Asia and Europe. Increased vector populations under hot and dry summer conditions, elevated stress levels in host plants, and accelerated nematode biological activity further accelerate the progression of the disease [35].

However, the effects of temperature increases are not uniform across all ecosystems and all PPN species. In alpine ecosystems of the Qinghai–Tibet Plateau, moderate warming increased the abundance of PPNs and showed positive correlations with genera such as *Helicotylenchus* and *Rotylenchus*, whereas higher warming levels reduced the abundance of some PPN genera including *Criconema* and *Scutellonema* [36]. Furthermore, it has been shown that temperature increases affect the distribution of soil nematode communities along elevation gradients and species differentiation processes [37].

One of the most important ecological consequences of temperature increase is the reshaping of nematode species geographic distribution boundaries. As shown in Figure 1, increased soil temperatures shorten nematode life cycles and enhance reproduction rates, while elevated CO_2_ levels affect plant defense mechanisms, thereby increasing infection and ultimately negatively impacting plant growth and yield.

Temperature-related climate change is also redefining the agricultural pest status of some PPN species. Climate scenario modelling conducted in the United Kingdom has shown that increasing soil temperatures may suppress *Globodera pallida* populations, but create more favourable conditions for *Globodera rostochiensis*, thereby increasing the spread and damage potential of this species in southern regions [38]. Similarly, it has been suggested that climate change could increase the risk of PPN in mountainous and high-altitude agricultural areas, with species such as *Radopholus similis* and *Pratylenchus goodeyi* becoming more prevalent in these areas [22]. Furthermore, some regional studies have revealed that *G. rostochiensis* has become an invasive pest in new geographical areas such as Russia due to the effects of global warming [39]. The effects of increasing temperatures on the development, reproduction rate, and geographical spread of PPNs are presented in Table 1, based on laboratory, controlled climate, and field studies.

Overall, as summarized in Table 1, increasing temperatures tend to accelerate nematode development and reproduction, promote the expansion of thermophilic species into new regions, and alter species dominance patterns, leading to shifts in pest status and distribution. These findings highlight that temperature is a key driver shaping both the biology and geographical spread of PPNs under climate change conditions.

### 3.2. Elevated Atmospheric CO_2_ and Plant–Nematode Interactions

Increased carbon dioxide (CO_2_) concentrations in the atmosphere cause significant changes in plant physiology, and these changes directly affect plant–parasite interactions [20]. Increased CO_2_ levels promote root development by increasing the rate of photosynthesis in plants; this, in turn, creates a wider infection area for PPNs [28]. Many studies have reported that plants growing under elevated CO_2_ conditions are more intensely infected, particularly by root-knot nematodes (*Meloidogyne* spp.). Furthermore, increased root biomass not only expands the feeding area but also creates a favorable microenvironment for PPN reproduction [22].

Increased atmospheric CO_2_ levels can affect plant defense mechanisms and indirectly alter PPN interactions. Under high CO_2_ conditions, some defense signaling pathways in plants show alterations, particularly in responses associated with jasmonic acid (JA) and salicylic acid (SA) [28]. Experimental studies have shown that rising CO_2_ suppresses JA-mediated plant defense responses and may, in some cases, lead to reduced resistance against nematodes [47]. For example, a study with *Meloidogyne incognita* reported that defense responses associated with the JA pathway were reduced under high CO_2_, and changes in plant defense responses against this parasite were observed. Furthermore, increased CO_2_ can also cause changes in the rhizosphere microbiome; since soil microorganisms affect surface chemical signals, root chemicals, and plant hormone signals, they can alter the behavior of nematodes in finding and infecting roots [28,48].

The effects of elevated CO_2_ levels on PPNs are complex and show species-specific differences. For example, Mueller et al. [49] reported that total PPN abundance decreased under high CO_2_ conditions, and that this decrease led to significant changes in the soil food web structure. Similarly, Neher and Weicht [50], in their study of pine forests, observed a decrease in the number of nematodes belonging to the genus *Aphelenchoides*, no significant change in the genus *Xiphinema*, and an increase in the populations of the genus *Longidorus*. These findings show that elevated CO_2_ can have different effects on nematode ecology at the species level. In general, disease formation in plants depends on the interaction of three main factors: plant susceptibility, pest virulence, and favorable environmental conditions [51]. Therefore, CO_2_ increase is considered a factor that directly increases the severity of PPN infections and poses a significant threat to future agricultural production.

The effects of elevated atmospheric CO_2_ conditions on the abundance and community structure of PPNs in different ecosystems, based on results from Free-air CO_2_ enrichment (FACE) and controlled environment experiments, are presented in Table 2. Overall, these studies indicate that elevated CO_2_ alters plant–nematode interactions by increasing root biomass and modifying plant defense responses, while its effects on nematode abundance and community structure are species-specific and ecosystem-dependent.

### 3.3. Altered Rainfall Regimes and Soil Moisture Dynamics

Rainfall variations and fluctuations in soil moisture are among the most important factors determining nematodes’ movement within the soil, their ability to find hosts, and their survival rates [12,62]. However, many nematode species can survive dry periods for long periods by entering a biological state of dormancy called anhydrobiosis and can become active again when moisture levels rise [6].

Excessive rainfall events can create different ecological pressures for PPNs. When oxygen levels drop due to waterlogged soil, mortality rates can increase, especially in oxygen-dependent (aerobic) nematode species [14]. However, following water withdrawal, when the soil begins to aerate again, some PPN species, particularly those capable of adapting to the post-stress environment, have been observed to exhibit more severe infection behavior against host plants [63]. Changes in rainfall regimes can indirectly shape the spread and reproduction potential of PPNs by affecting not only soil moisture directly, but also soil structure, microbial balance, and plant root defense responses [31]. Consequently, it is predicted that precipitation irregularities caused by climate change will significantly alter the regional distribution of PPN populations in the future. Species-specific and regional effects of changes in precipitation regimes and soil moisture dynamics on PPN populations are presented in Table 3 based on long-term field manipulations and observational studies. Overall, these findings indicate that soil moisture fluctuations play a dual role by both suppressing nematode survival under extreme conditions such as waterlogging and enhancing dispersal and infection potential under favorable moisture levels, with outcomes varying depending on species and environmental context.

### 3.4. Extreme Climate Events and Nematode Population Responses

Global warming has increased the frequency of extreme climate events such as heat waves, floods, sudden frost events, and forest fires [71,72]. While all these events severely impact soil ecosystems, they also cause dramatic changes in PPN populations. Fires can weaken nematode habitats by reducing organic matter in the topsoil; however, it has been reported that some species recover very quickly after a fire and fill the vacant ecological space, forming larger populations [15,73].

In connection with climate change, an increase in the frequency of extreme weather events such as heat waves, droughts, heavy rainfall, forest fires, storms, and hail has been observed, threatening food security and ecosystem services on a global scale [74]. Nematodes have the ability to survive in such harsh environments by developing various morphological and physiological adaptations to environmental stress conditions [25].

Flooding can create hypoxic (oxygen-depleted) environments in the soil, increasing the mortality of aerobic PPN species. However, some species possess adaptations that allow them to temporarily withstand low oxygen conditions, enabling them to multiply rapidly in the post-flood period [30]. Sudden heat waves can also facilitate PPN infections by lowering the defences of plants weakened by stress. Decreased plant resistance can result in population increases, particularly of *Meloidogyne* and *Heterodera* species [43]. Therefore, the increase in extreme events has become one of the most critical factors determining the future of PPN ecology [73].

## 4. Plant Defense Responses, Nematode Biology, and Ecosystem-Level Interactions

Under climate change conditions, the defense responses developed by plants against PPNs show significant changes at both the structural and molecular levels [22]. Within the scope of structural defense, thickening of root cell walls, increased lignin accumulation, and strengthening of mechanical barriers in epidermal tissues form the first line of defense [75]. However, during PPN infection, plants activate defense signaling through hormones such as salicylic acid (SA), jasmonate (JA), and ethylene (ET), thereby regulating the expression of defense-related genes [76].

Under climate change conditions, PPNs have developed various evolutionary strategies to render their hosts’ defense mechanisms ineffective. Economically important species such as *Meloidogyne* and *Heterodera* facilitate infection by secreting cell wall-degrading enzymes such as cellulase, pectinase, and protease during entry into root tissues [77]. In addition, they produce specific effector proteins to suppress the host plant’s immune responses and trigger cellular reprogramming [75]. These effectors promote the formation of specialized feeding structures such as giant cells or syncytia, enabling nematodes to feed for extended periods [78]. It has been reported that increasing temperatures can accelerate the infection process by enhancing these enzymatic activities and effector activity [31].

One of the significant consequences of global warming is the changes in the structure of rhizosphere microbial communities [79]. Microorganisms play a critical role in plant–nematode interactions; some bacterial and fungal species produce toxic compounds that break down nematode eggs, while others can suppress PPN infections by stimulating plant defense responses [80]. However, increasing temperatures and CO_2_ levels can lead to a decline in beneficial microbial communities and the dominance of opportunistic microorganisms [79]. This situation allows nematodes to find more favourable conditions in the soil environment and increases the risk of infection [73].

**Figure 2:**
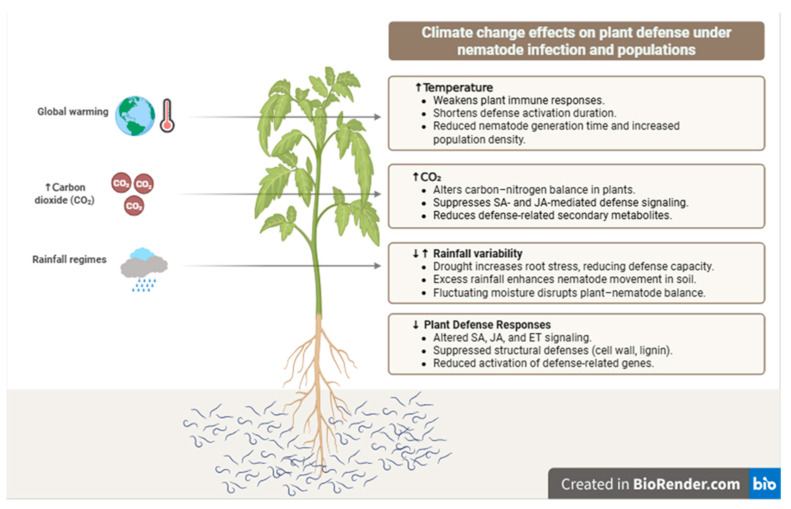
Climate change-driven modulation of plant defense responses and plant-parasitic nematode populations.

Overall, climate change affects the balance between plant defense mechanisms, nematode biology, and soil ecosystems in a multidimensional and species-specific manner [78]. As shown in Figure 2, climate driven factors, including rising temperatures, elevated CO_2_ levels and altered precipitation regimes, can disrupt hormonal signaling networks, impair defense coordination and ultimately reduce plant resistance against PPNs.

## 5. Climate-Adaptive Strategies for Managing Plant-Parasitic Nematodes

Climate change is significantly altering the effectiveness and predictability of traditional approaches used in the management of PPNs [22]. Rising temperatures, changing rainfall patterns, and increasing atmospheric CO_2_ levels not only reshape nematode population dynamics but also directly influence the performance of management strategies by affecting soil conditions, plant responses, and nematode biology [81]. In this context, integrated pest management (IPM) offers a flexible framework that can adapt to these climate-driven changes by addressing environmental sustainability, economic feasibility, and biological efficacy simultaneously [82]. The IPM approach is based on the simultaneous application of different control strategies in a manner compatible with climatic conditions, thereby reducing dependence on a single control method [83,84].

Cultural control methods stand out as one of the key components of climate-compatible nematode management [85]. Practices such as crop rotation, solarization, organic matter applications, and green manuring contribute to suppressing PPN populations by disrupting host–parasite continuity and regulating soil biological activity [82]. These practices are directly influenced by climate drivers; for instance, increasing temperatures enhance the effectiveness of soil solarization, while changes in rainfall patterns and soil moisture affect the success of crop rotation and organic amendments. It has been reported that soil solarization has become more effective in many regions, particularly due to the effects of global warming, and that nematodes and their eggs can be significantly inactivated when soil temperatures reach 45–55 °C [86,87]. However, since the effectiveness of cultural practices varies depending on soil type, crop pattern, and regional climatic conditions, it is crucial to adapt these methods to local conditions [88].

While chemical control continues to be a supportive element within IPM, it is increasingly used in a limited and controlled manner due to environmental risks, residue problems, and rising application costs [82]. In particular, rising temperatures can accelerate the degradation of nematicides and shorten their efficacy period, thereby reducing their effectiveness under field conditions [89]. This highlights the need to integrate chemical control with other strategies under changing climate conditions [82].

Biological control plays a critical role in climate-resilient nematode management due to its environmentally friendly nature and long-term suppression potential [90]. Microorganisms such as *Pochonia chlamydosporia*, *Pasteuria penetrans*, *Trichoderma* spp., and *Bacillus* spp., as well as nematode-trapping fungi (*Arthrobotrys* spp.), are effective in reducing PPN populations [91,92,93,94]. However, climate-related factors such as temperature fluctuations and soil moisture variability directly affect the survival, reproduction, and infectivity of these biological control agents, thereby influencing their field performance. Therefore, selecting climate-resilient biological agents is becoming increasingly important [95,96].

The use of resistant plant varieties is considered one of the most effective and economical control methods against PPNs [97]. However, increasing temperatures, as a key climate driver, can limit the effectiveness of resistance genes, with resistance breakdown occurring under conditions above 32 °C [98]. This demonstrates that plant resistance is also sensitive to climate change and highlights the need for developing heat-stable resistant varieties.

Nowadays, the application of biochar as a soil amendment has attracted increasing attention. Biochar improves soil physical, chemical, and biological properties, helping to buffer the effects of climate change such as fluctuating temperature and moisture conditions [99]. It enhances soil structure, water retention, and nutrient availability, while also promoting beneficial microbial communities that suppress nematode populations [100,101]. Through these mechanisms, biochar contributes to reducing PPN pressure under climate change scenarios.

The success of IPM applications under climate change conditions is directly related to monitoring and early warning systems [102]. The integration of sensor-based technologies that monitor soil temperature, moisture levels, and nematode density with climate models enables the early detection of climate-driven PPN risk [103]. These systems allow producers to implement timely and targeted management strategies in response to climate variability, thereby improving the overall effectiveness of nematode control [104].

**Figure 3:**
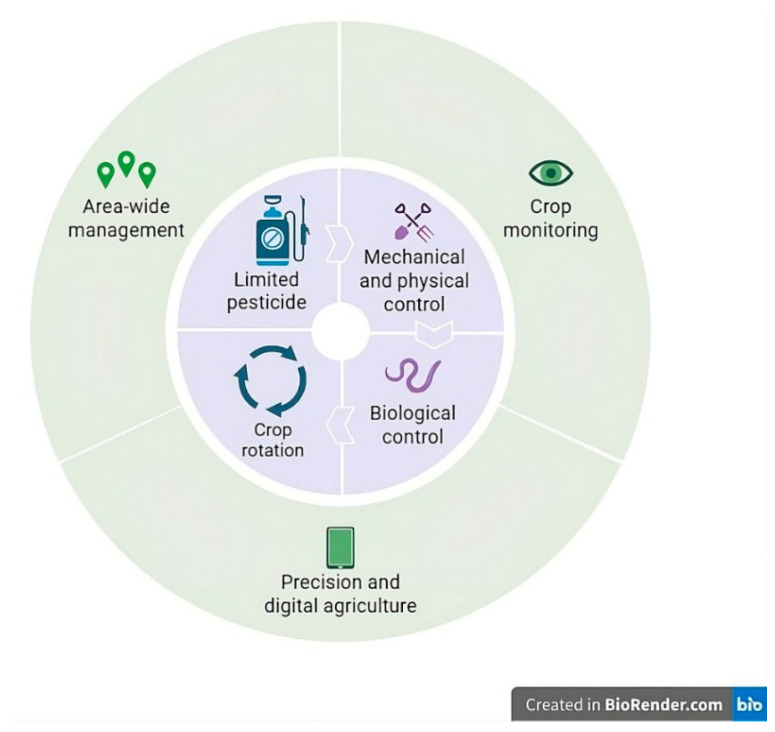
Climate-adaptive integrated management of plant-parasitic nematodes, highlighting cultural, biological and digital agricultural systems.

As shown in Figure 3, climate-adapted IPM strategies combine area-wide management, crop monitoring, precision and digital agriculture, crop rotation, biological control, mechanical and physical control, and limited pesticide use to effectively manage PPNs. In general, with the increasing effects of climate change, the management of PPNs requires IPM approaches that are sensitive to regional conditions and flexible, taking environmental variables into account, rather than conventional practices. The climate-compatible integration of cultural, biological, and technological components is emerging as a key strategy for reducing PPN-related risks.

## 6. Conclusions and Future Prospects

This review highlights that global warming has decisive and multidimensional effects on the biology, population dynamics, and geographic distribution of PPNs. Rising temperatures shorten the development period of nematodes, increasing the number of generations within a production season and facilitating the spread of many economically important species to higher latitudes and altitudes. This process leads to an increase in the severity of nematode-related damage and greater losses in agricultural production in terms of yield and quality.

However, the responses of PPNs to climate change drivers are not uniform across studies. Variability and, in some cases, contradictory findings indicate that these responses are strongly context-dependent. Differences in species-specific traits, such as thermal tolerance, life cycle strategies, and host specificity, as well as variations in experimental design, environmental conditions, and soil characteristics, contribute to these inconsistencies. In addition, discrepancies between controlled environment experiments and field-based observations further complicate interpretation, highlighting the need for more integrative and multi-factorial approaches.

The effects of global warming are not limited to nematode populations; they also cause significant changes in the physiology and defense mechanisms of host plants and in the functioning of soil ecosystems. Rising temperatures, elevated atmospheric CO_2_ levels, irregular rainfall patterns, and fluctuations in soil moisture make plants more susceptible to nematode infections while further increasing the complexity of plant–nematode–soil interactions. This situation leads to the dominance of certain PPN species and reshapes regional risk patterns.

These findings indicate that current nematode control strategies are not always sufficient under climate change conditions. In particular, the reduced effectiveness of chemical control at high temperatures and its environmental risks necessitate the adoption of more sustainable and climate-resilient approaches. In this context, integrated nematode management approaches combining cultural practices, biological control agents, resistant plant varieties, and limited chemical interventions when necessary, provide an effective framework that can adapt to changing climate conditions.

Future studies should address the effects of climate change on PPNs by species and region, and focus on long-term field studies that evaluate the simultaneous effects of factors such as temperature, CO_2_, and soil moisture. Furthermore, the development of biological control agents that are more resistant to climate variables, the identification of resistance genes that maintain their effectiveness at high temperatures, and the integration of early warning systems into agricultural production will play a critical role in reducing nematode-related risks.

In conclusion, global warming is reshaping the ecology and agricultural damage levels of PPNs; this situation requires the reassessment of nematode management strategies based on approaches that centre on climate change, are sensitive to regional conditions, and are sustainable. Climate-adaptive nematode management will be an indispensable component in ensuring sustainable agricultural production and global food security in the future.

## Figures and Tables

**Table 1:** Temperature responses of plant-parasitic nematodes under experimental and field-based warming conditions.

Cropping System	Experimental System	Nematode	Temperature Response	Country	Reference
Laboratory growth test	Controlled environment developmental assays	*Meloidogyne* spp. (incl. *M. enterolobii*, *M. floridensis*)	Accelerated development at higher temperatures	Laboratory-based	[32]
Potato	Controlled-climate growth chamber experiments	*Globodera rostochiensis*, *G. pallida*	Population increase in *G. rostochiensis*; limited response in *G. pallida*	United Kingdom	[38]
Pine forest	Long-term ecosystem observation and tree-based sampling	*Bursaphelenchus mucronatus*, *B. xylophilus*	Higher occurrence and broader distribution under warmer conditions	Switzerland	[40]
Coffee plantation	Climate-based simulation model	*Meloidogyne incognita*	Increased disease risk under elevated temperature scenarios	Brazil	[41]
Laboratory incubation	Controlled laboratory incubation across temperature gradients	*Rotylenchulus reniformis*	Embryogenesis maintained at elevated temperatures with population-specific optima	USA	[42]
Carrot	Temperature-driven simulation experiment	*Heterodera carotae*	Increased juvenile density and egg production	Italy	[43]
Potato	Soil-temperature controlled hatch and biological assays	*Globodera rostochiensis*, *G. pallida*	Species-specific differences in hatch response to warming	Europe	[44]
Grassland	Active field warming through infrared radiation	*Aphelenchoides* spp., *Paratylenchus* spp., *Pratylenchus* spp., *Rotylenchus* spp.	Daytime warming exerted stronger suppressive effects than nighttime warming	China	[45]
Laboratory growth test	Controlled soil-temperature assays (direct & indirect heat exposure)	*Rotylenchulus reniformis*, *Meloidogyne floridensis*	Reduced reproduction with species-specific survival and virulence responses	USA	[46]

**Table 2:** Effects of elevated CO_2_ on plant-parasitic nematodes across different ecosystems and experimental systems.

Cropping System	Experimental System	Nematode	CO_2_-Related Response	Country	Reference
Pasture/grassland	Natural soil venting (CO_2_ enrichment)	*Pratylenchus* spp.	Higher population levels associated with CO_2_ enrichment	New Zealand	[6]
Rice and wheat rotation	Free-air CO_2_ enrichment (FACE)	*Filenchus* spp., *Psilenchus* spp., *Hirschmanniella* spp., *Pratylenchus* spp.	Nematode abundance unchanged, while community diversity increased	China	[47]
Grassland	Factorial field experiment (elevated CO_2_ × warming)	Plant-feeding nematodes	Weak CO_2_-only effect; shift under CO_2_ × warming	USA	[49]
Ryegrass/white clover	Controlled environment	*Dorylaimus* spp., *Trichodorus* spp.	Increased nematode abundance under elevated CO_2_	New Zealand	[52]
Grassland	Open-top chambers	*Meloidogyne* spp., *Neopsilenchus* spp., *Paratylenchus* spp., *Gracilacus* spp., *Xiphinema* spp.	Overall increase in nematode abundance	USA	[53]
Grassland	Free-air CO_2_ enrichment (FACE)	*Tylenchus* spp., *Longidorus* spp.	Positive population response to elevated CO_2_	New Zealand	[54]
Grassland	Screen-aided CO_2_ control	Not specified	No significant change in nematode abundance	Switzerland	[55]
Grassland	Free-air CO_2_ enrichment (FACE)	Not specified	Transient increase followed by a return to baseline levels	Germany	[56]
Rice–wheat rotation	Free-air CO_2_ enrichment (FACE)	*Psilenchus* spp., *Hirschmanniella* spp., *Filenchus* spp., *Tylenchus* spp.	Increased abundance under elevated CO_2_ conditions	China	[57,58]
Grassland	Open-top chambers	*Heterodera* spp., *Meloidogyne* spp., *Longidorus* spp., *Trichodorus* spp., *Criconema* spp., *Tylenchus* spp., *Tylenchulus* spp.	No overall response, despite increased abundance of anguinid root feeders	USA	[59]
Sugar beet/wheat rotation	Free-air CO_2_ enrichment (FACE)	Not specified	Increased nematode abundance under elevated CO_2_	Germany	[60]
Rice paddy field	Elevated CO_2_ + canopy warming (field)	*Pratylenchus*	CO_2_ increases abundance, while warming reduces diversity.	China	[61]

**Table 3:** Effects of altered precipitation on plant-parasitic nematodes.

Cropping System	Experimental System	Nematode	Precipitation Response	Country	Reference
Grassland/heathland	Summer rain exclusion (field)	Not specified	Community shift toward long-lived taxa	Denmark	[64]
Desert ecosystem	Long-term precipitation manipulation	Not specified	Increased abundance under higher precipitation	USA	[65]
Grasslands and cereal systems	Field-based observational sampling	*Meloidogyne minor*, *Heterodera* spp., *Pratylenchus* spp.	Higher abundance with rainfall variability	Northern Ireland	[66]
Grassland	Field precipitation manipulation	Not specified	Drought increased root-feeders at mesic site	USA	[67]
Blue grama grass (*Bouteloua gracilis*)	Greenhouse soil-moisture experiment	Not specified	Water stress intensified root herbivory	USA	[68]
Desert grassland to tallgrass prairie	Field precipitation manipulation	*Ditylenchus* spp., *Hemicycliophora* spp., *Hoplolaimus* spp., *Pratylenchus* spp., *Rotylenchus* spp., *Subanguina* spp.	Endoparasites favored by higher rainfall	USA	[69]
Arid to mesic grasslands	Long-term precipitation manipulation	*Paratylenchus* spp., *Helicotylenchus* spp., *Hoplolaimus* spp., *Trichodorus* spp., *Xiphinema* spp., *Longidorus* spp.	Opposite responses across moisture gradients	USA	[70]

## Data Availability

No new data were created or analyzed in this study.

## Abbreviations

The following abbreviations are used in this manuscript:

PPN	Plant-parasitic nematode
CO_2_	Carbon dioxide
FACE	Free-air CO_2_ enrichment
IPM	Integrated Pest Management
